# The PD-(D/E)XK superfamily revisited: identification of new members among proteins involved in DNA metabolism and functional predictions for domains of (hitherto) unknown function

**DOI:** 10.1186/1471-2105-6-172

**Published:** 2005-07-12

**Authors:** Jan Kosinski, Marcin Feder, Janusz M Bujnicki

**Affiliations:** 1 Laboratory of Bioinformatics and Protein Engineering, International Institute of Molecular and Cell Biology, Trojdena 4, PL-02-109 Warsaw, Poland

## Abstract

**Background:**

The PD-(D/E)XK nuclease superfamily, initially identified in type II restriction endonucleases and later in many enzymes involved in DNA recombination and repair, is one of the most challenging targets for protein sequence analysis and structure prediction. Typically, the sequence similarity between these proteins is so low, that most of the relationships between known members of the PD-(D/E)XK superfamily were identified only after the corresponding structures were determined experimentally. Thus, it is tempting to speculate that among the uncharacterized protein families, there are potential nucleases that remain to be discovered, but their identification requires more sensitive tools than traditional PSI-BLAST searches.

**Results:**

The low degree of amino acid conservation hampers the possibility of identification of new members of the PD-(D/E)XK superfamily based solely on sequence comparisons to known members. Therefore, we used a recently developed method HHsearch for sensitive detection of remote similarities between protein families represented as profile Hidden Markov Models enhanced by secondary structure. We carried out a comparison of known families of PD-(D/E)XK nucleases to the database comprising the COG and PFAM profiles corresponding to both functionally characterized as well as uncharacterized protein families to detect significant similarities. The initial candidates for new nucleases were subsequently verified by sequence-structure threading, comparative modeling, and identification of potential active site residues.

**Conclusion:**

In this article, we report identification of the PD-(D/E)XK nuclease domain in numerous proteins implicated in interactions with DNA but with unknown structure and mechanism of action (such as putative recombinase RmuC, DNA competence factor CoiA, a DNA-binding protein SfsA, a large human protein predicted to be a DNA repair enzyme, predicted archaeal transcription regulators, and the head completion protein of phage T4) and in proteins for which no function was assigned to date (such as YhcG, various phage proteins, novel candidates for restriction enzymes). Our results contributes to the reduction of "white spaces" on the sequence-structure-function map of the protein universe and will help to jump-start the experimental characterization of new nucleases, of which many may be of importance for the complete understanding of mechanisms that govern the evolution and stability of the genome.

## Background

The PD-(D/E)XK superfamily of Mg^2+^-dependent nucleases groups together protein domains initially identified in structurally characterized type II restriction enzymes (REases) (reviews: [1,2]) and later found in diverse enzymes involved in DNA replication, repair, and recombination, including phage λ exonuclease [3], bacterial enzymes exerting ssDNA nicking in the context of methyl-directed and very-short-patch DNA repair: MutH [4] and Vsr [5], Tn7 transposase TnsA [6], archaeal Holliday junction resolvases (AHJRs) Hjc/Hje [7-9], phage T7 endonuclease I [10], the XPF/Rad1/Mus81 family of nucleases that cleaves branched structures generated during DNA repair, replication, and recombination [11], and RecB nuclease [12].

**Figure 1:**
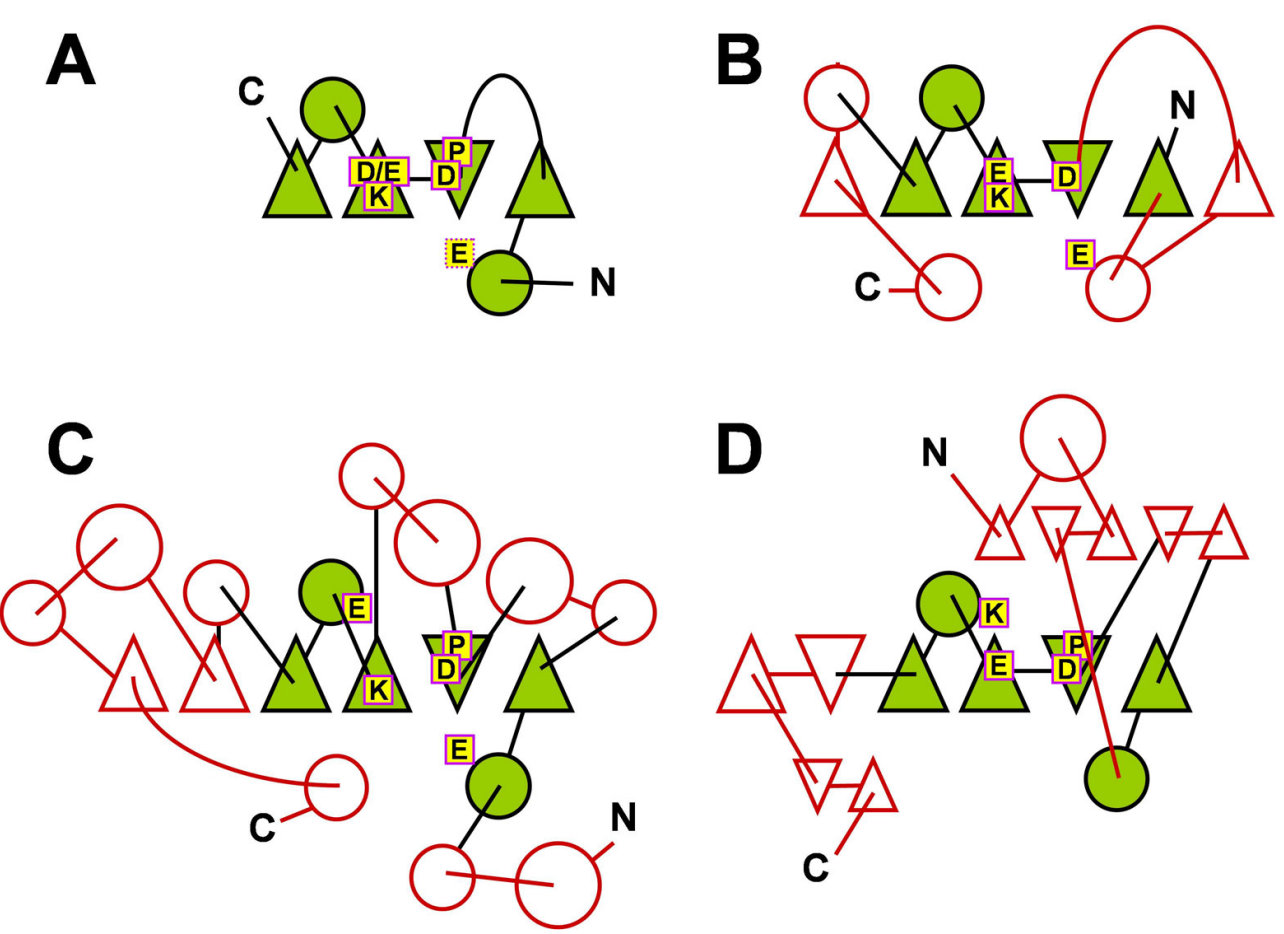
**A) Conserved topology of the common core and the most typical architecture of the active site in PD-(D/E)XK nucleases, B) Topological variation of the common fold found in the nuclease XPF (1j24), C) Extension of the common core and migration of the carboxylate residue (e.g. in NgoMIV, 1fiu), D) Different extension of the common core and migration of the lysine residue (e.g. in Tt1018, 1wjd). **Helices are shown as circles, strands are shown as triangles (the orientation – up or down indicates the direction and the parallel/antiparallel character). The common core is shown in green/black, the variable elaborations are shown in red/white. The most common catalytic residues (known or putative) are indicated by letters in yellow boxes (E, D, and K), structurally important, semi-conserved P residue is also indicated.

All members of the PD-(D/E)XK superfamily share a common structural core, comprising a mixed β-sheet of 4 strands flanked on both sides by α-helices [1,2,13](Figure 1). It serves as a scaffold for a weakly conserved active site, typically including two or three acidic residues (Asp or Glu) and one Lys residue, which together form the hallmark bipartite catalytic motif (P)D...X_n_.(D/E)X K (where X is any amino acid). It was found that some members of the PD-(D/E)XK superfamily developed different variants of the active site, in which the acidic residues or the lysine have been replaced by Asn or Gln (Lys can be also replaced by an acidic side chain) [14-16], or in which their side-chains have "migrated" to another region of the polypeptide in a way that only the spatial orientation of functional groups in the active site is maintained, but their sequence is not conserved [17-21] (Figure 1). This variability makes it difficult to identify the active site in PD-(D/E)XK nucleases solely based on sequence comparisons and usually requires analysis of the three-dimensional structures (e.g. obtained by comparative modeling techniques). It should be mentioned that the mechanistic/catalytic role of the active site sidechains is not fully elucidated even in the well-characterized members with known structure. Moreover, many of PD-(D/E)XK nucleases contain elaborations of the common fold in the form of large insertions and terminal extensions that form additional subdomains, usually involved in oligomerization or DNA-binding. These regions often contain regular elements of secondary structure and sometimes constitute the majority of the protein, making the detection of the true core a challenging task for protein structure prediction methods (reviews: [2,22]).

We have previously used the comparisons of sequence profiles to detect novel members of the PD-(D/E)XK superfamily [16,18,19,23-25], as well as applied the sequence-structure threading to map the sequences of REases onto the crystal structures of known members [21,26-28]. However, both approaches have shortcomings: the profile-profile analysis is unable to find very remote relationships when families include only a few members (as is often the case in the PD-(D/E)XK superfamily) or if the compared proteins contain large unrelated insertions, and the threading analysis works only if an experimentally solved structure is available for one of the potential homologs. Therefore, we decided to use an intermediate approach, in which the compared sequence profiles are combined with the structural information, which can be obtained either from the crystal structure (if it is available for one of the members of the considered families) or predicted by bioinformatics methods, thereby allowing protein fold recognition without the explicit reference to an experimentally solved protein structure. We used the recently developed method HHsearch that allows comparison of sequence alignments together with secondary structures represented as Hidden Markov Models (HMM) [29] to compare the library of PD-(D/E)XK nuclease families obtained previously [23] with the set of domains in the Clusters of Orthologous Groups (COG and KOG)[30] and PFAM [31] databases. Our aim was to identify the previously unknown PD-(D/E)XK domains in families of proteins that are either completely uncharacterized, or whose function is known (or predicted), but the structure and phylogenetic relationships are unclear.

## Results

### Identification of new candidate PD-(D/E)XK nucleases with profile HHM searches

In order to carry out a systematic search for new PD-(D/E)XK nucleases, we prepared a set of multiple sequence alignments corresponding to previously identified families [18,23,32](see Methods). For each family we generated a profile HMM that included the sequences and predicted secondary structure (see Methods). These profile HMMs were compared with HHsearch [29] to a database of profile HMMs corresponding to multiple sequence alignments obtained from the COG, KOG, and PFAM databases, also with predicted secondary structures (see Methods for details). It is noteworthy, that searches initiated with most of the known PD-(D/E)XK nucleases in the query dataset identified families containing sequences of other known PD-(D/E)XK nucleases, thereby validating our approach and providing useful threshold values for confident identification of true members of PD-(D/E)XK superfamily. For instance, AtF16A14.4 profile detected herpesvirus alkaline exonuclease (pfam01771) with the e-value of 10^-3^, Pfu-HJR detected Mrr with the e-value of 10^-2^, YcjD detected Vsr with the e-value of 10^-5^, etc. It must be noted, that some of the "best hits" to true positives exhibited poor scores, e.g. Mrr detected HJR (pfam01870) with the e-value of 1.2. As expected, our HHM-HHM comparisons revealed also numerous other potential homologs, not included in the query dataset. Based on these results, we generated a preliminary list of candidate new PD-(D/E)XK subfamilies. For further analysis, we retained only such proteins, which have not been reported as members of the PD-(D/E)XK superfamily in earlier analyses carried out by ourselves or other groups (e.g. [32]).

The preliminary candidates for novel PD-(D/E)XK nucleases were initially validated by reciprocal HHsearches against the database comprising both the initial query profile HMMs as well as all the other COG, KOG, and PFAM profile HMMs. This search confirmed that for most of the candidate families, the closest homologs are either among the bona fide PD-(D/E)XK enzymes or among other preliminary candidates. It also revealed additional families, which were similar to the first set of candidates, but not to the original PD-(D/E)XK queries, suggesting that they may be more remote members of the superfamily. In reciprocal searches, a limited number of profile HMMs showed significant relationship to other families, unrelated to PD-(D/E)XK enzymes – such candidates were regarded as false positives and were not further analyzed. Further, each candidate family was analyzed by fold-recognition methods to evaluate its compatibility with the known PD-(D/E)XK structures (or detect cases, where some other, unrelated structure appeared to be a better template). Finally, preliminary comparative models were built for the parts of the sequence aligned to the template structures and the sequence conservation was analyzed in the structural context to detect potential active site residues. Altogether, our analysis revealed previously unknown membership of 14 families and one "orphan" protein (herein predicted to be a new restriction enzyme) in the PD-(D/E)XK superfamily of nucleases (Table 1).

**Table 1:** New members of the PD-(D/E)XK superfamily Columns I and II contain accession numbers of Pfam and COG database entries corresponding to protein families analyzed in this work. Typically, COGs contain only subsets of seqeunces from the corresponding Pfam entries. Previously described molecular and/or cellular function and the newly predicted function is shown. * indicates only a general functional prediction based on the fact that a given protein family is found to possess a nuclease domain with a seemingly complete set of catalytic residues.

**PFAM**	**COG**	**representative**	**closest homolog**	**other domains**	**known function**	**predicted function**
02646	1322	*E. coli *RmuC	McrC	transmembrane helix, coiled-coil regions, unknown alpha/beta domain	limiting inversions at short-inverted repeats	cleaves DNA structures arising during recombination of short- inverted repeats
06054	4469	*S. pneumoniae *CoiA	COG 4636	unknown C-terminal domain	DNA uptake process and recombination	degradation of one DNA strand during DNA uptake
03749	1489	*E. coli *SfsA	YraN	OB-fold N-terminal domain	sugar fermentation stimulation	DNA cleavage*
-	2143 (KOG)	*H. sapiens *KIAA1018	AHJR	TPR-like domain, Rad18-like CCHC zinc finger, uknown 300aa domain	unknown	DNA repair, protein binding
06250	4804	*E. coli *YhcG	YraN	~200aa unknown N-terminal domain	unknown	DNA cleavage*
06356	-	Phage phi ETA orf25	TnsA or T7 EndoI	none	unknown	degradation of the host DNA upon lytic infection, production of recombinogenic fragments
-	5482	*A. tumefaciens *AGRL2570	AHJR	C-terminal domain with wHTH fold	unknown	DNA binding, DNA cleavage
06190	-	Phage PSA gp51	AHJR	none	unknown	recombinase
-	1395	*P. abyssi *PAB2104	AHJR	second inactive AHJRlike domain, HTH_3 domain	unknown	DNA cleavage or nicking*
-	4127	*S. typhimurium *STM4490	Mrr	putative inactivated nuclease domain	unknown	REase
	4741	*T. volcanium *TVN1166	YraN	N-terminal membrane helix	unknown	membrane-associated guardian against foreign DNA
06319	5321	*A. tumefaciens *AGR_C_8	AHJR	none	unknown	DNA cleavage*
05626	3372	*P. abyssi *PAB1046	AHJR	unknown ~250aa N-terminal domain	unknown	DNA repair or recombination
-	-	Phage T4 gp4	TnsA or T7 EndoI	none	important for the phage T4 head assembly process [52]	determination if DNA is packed properly into the phage head
-	-	*P. aerophilum *PAE1662	AHJR	none	unknown	type II REase

## Discussion

### COG1322/pfam02646 (RmuC family)

**Figure 2:**
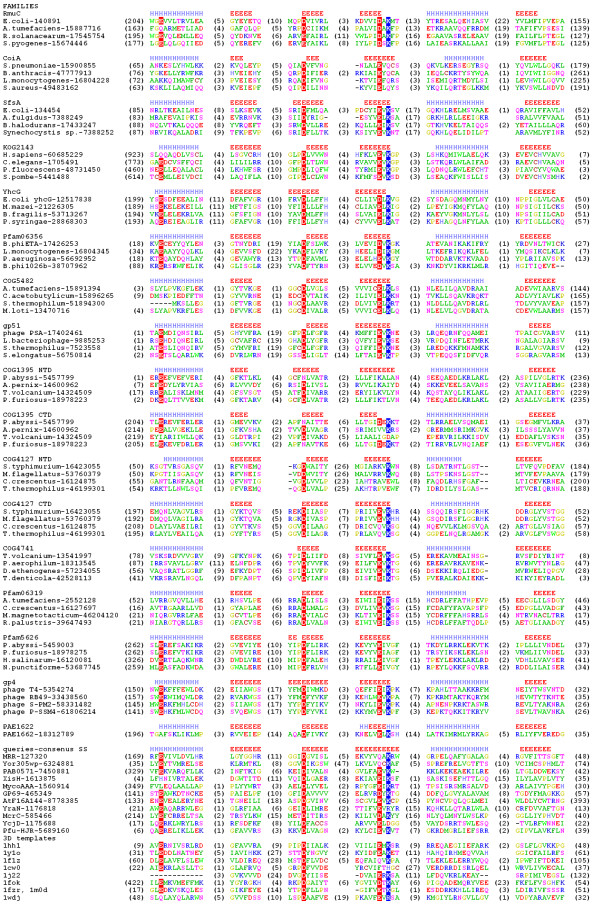
**Multiple sequence alignment of selected representatives of proteins families predicted to belong to the PD-(D/E)XK superfamily**. The selection of representative sequences includes members of all families analyzed in this article (see Table 1). Amino acids are colored according to the physico-chemical properties of their side-chains (negatively charged: red, positively charged: blue, polar: magenta, hydrophobic: green). The putative catalytic Asp/Glu and Lys/Arg residues are highlighted. The variable termini and insertions are not shown. The number of omitted residues is indicated in parentheses. Elements of secondary structure (helices and strands predicted individually for each family) are indicated by H and E, respectively.

COG1322/pfam02646 is represented by the RmuC protein from *Escherichia coli*, which has been implicated in limiting inversions at short-inverted repeats [33]. It has been speculated that RmuC exhibits "limited homology" to human Rad50 protein, centrosome protein pericentrin, nuclear mitotic apparatus proteins and the SbcC proteins, and therefore it may be a structural protein that protects DNA against nuclease action or be itself involved in DNA cleavage at the regions of DNA secondary structure [33]. Our secondary structure predictions revealed that RmuC contains an N-terminal transmembrane helix (aa 1–25), coiled-coil structures (aa 25–200 and ~350–420), a globular α/β domain (aa 200–360), and a disordered C-terminus (aa 420–475). The profile HMM analysis confidently identified a relationship of the central globular α/β domain of RmuC and its homologs to the catalytic domain of the McrC nuclease [23,34](P-value 10^-4^). This prediction was also confirmed by the fold-recognition analysis, which identified the catalytic domain of REase FokI as the best modeling template, albeit with relatively low scores (mGenTHREADER: 0.4666, SAM-T02: 0.23). Analysis of the multiple sequence alignment (Figure 2) reveals that proteins from the RmuC/COG1322/pfam02646 family exhibit a hallmark PD-(D/E)XK motif associated with the characteristic pattern of predicted secondary structures, which strongly suggests that RmuC is a nuclease that may cleave DNA structures arising during the recombination of short-inverted repeats and thereby thwart the inversion of the internal sequence. We suspect that the initial predictions of homology between RmuC and proteins involved in the structural maintenance of chromosome (Rad50 and SbcC) [33] was due to a spurious similarity in the regions of low sequence complexity, e.g. coiled coils.

### COG4469/pfam06054 (CoiA family)

COG4469/pfam06054 is represented by the CoiA protein from *Streptococcus pneumoniae*, which has been implicated in the DNA uptake process and recombination, but without any clues as to the molecular mechanism of its action [35]. The profile HMM analysis identified a relationship (P-value 10^-2^) between the central region of CoiA (aa 56–209) and COG4636, a family of predicted nucleases abundant in Cyanobacteria [21]. This prediction was also confirmed by the fold-recognition analysis, which aligned this domain to the recently solved structure of the COG4636 member (Structural Genomics target Tt1808 from *Thermus thermophilus *Hb8, 1wdj in the PDB, annotated as "to be published") (top matches in FFAS, score -11.0, and mGenTHREADER, score 0.369) and the structure of the Hef nuclease [11](1j22 in the PDB, INBGU top match, score 8.38), which both belong to the PD-(D/E)XK superfamily. While the template structures exhibit unorthodox configurations of catalytic residues (migration of the Lys residue in COG4636 members and topological crossover and migration of the Glu residue in Hef and its homologs), members of the CoiA/COG4469/pfam06054 family exhibit a more typical PD-EXQ variant of the active site, which has been observed in REase BglII [15]. Additionally, CoiA and its homologs possess a conserved Arg residue at the same position as the swapped Lys residue in COG4636. It will be interesting to determine if this residue is involved in substrate binding or catalysis. We hypothesize that in competent pneumococcus cells, the CoiA nuclease degrades one strand of the double-stranded DNA, while the other strand is imported inside the cell. Alternatively, CoiA may be involved in the incorporation of the foreign DNA into the chromosome, but this process is more likely to be carried out by the general homologous recombination machinery, such as RecA.

### COG1489/pfam03749 (SfsA family)

COG1489/pfam03749 is represented by the SfsA protein from *E. coli*, which has been implicated in sugar fermentation stimulation by the so far unknown mechanism and was shown to non-specifically bind to DNA [36]. The profile HMM analysis confidently identified a relationship (P-value 10^-5^) between the C-terminal domain of SfsA (aa 80–234) and the YraN family of previously predicted endonucleases [23]. This finding was confirmed by the fold-recognition analysis, which confidently identified the Vsr nuclease, a member of the PD-(D/E)XK superfamily (1vsr and 1cw0 in the PDB [37]) as the best template for the sequence of the SfsA C-terminal domain (scores: INBGU: 17.65, mGenTHREADER: 0.428, FUGUE: 3.4). Interestingly, while the Vsr nuclease exhibits a highly unorthodox active site, with the (D/E)XK half-motif replaced by FXH, SfsA and its homologs (including the YraN family) exhibit a typical version of the nuclease catalytic motif, ID-EVK. The fold-recognition analysis revealed also that the N-terminal domain of SfsA (aa 1–80) is a member of the OB-fold superfamily of nucleic acid binding proteins [38]. Thus, both domains of SfsA are likely to be involved in DNA binding, while the C-terminal domain is likely to possess an unforeseen nuclease activity. It will be interesting to determine whether the N-terminal or C-terminal domains or both are important for the activity of sugar fermentation stimulation and how does this activity relate to the predicted nuclease function. Or perhaps SfsA could be a hydrolase involved in another type of reaction than its homologs from the PD-(D/E)XK superfamily? Deletion mutagenesis based on our prediction and site-directed mutagenesis of the PD-(D/E)XK active site may help to address these questions.

### KOG2143 (KIA001018 family)

KOG2143 groups together 4 large (>1000 aa) eukaryotic proteins, including the KIA001018 protein from *H. sapiens *(PMID: 10231032), which has been annotated as a DNA binding protein engaged in DNA repair, probably due to a presence of an easily identified Rad18-like CCHC zinc finger in the N-terminus [39]. However, no suggestions concerning the mechanism of its action have been made. PSI-BLAST searches revealed that KOG2143 has homologs in other Eukaryota (plants and fungi) as well as in several bacteria (shorter versions, about 550 aa). The C-terminal domain (aa 935–1016 in KIA001018) shows significant similarity to HJR families (HHsearch P-values ranging from 10^-3 ^to 10^-5^) and exhibits the orthodox version of the PD-(D/E)XK motif, perfectly conserved in all members of KOG2143 as well as in their prokaryotic relatives. The fold-recognition analysis identified AHJR (1gef) as one of the possible templates, but with low confidence (mGenTHREADER, score 0.406). The analysis of predicted secondary structures and the preliminary tertiary model of KIA001018 suggest that the putative nuclease domain in KOG2143 and related proteins corresponds to the minimal core of the PD-(D/E)XK fold, i.e. only 4 β-strands and 2 α-helices [40] (Figure 1). Combination of reciprocal HHsearches and fold-recognition carried out for the remaining part of the KIA001018 sequence revealed that KOG2143 members possess a long (300–400 aa) α-helical domain, most probably comprising 5–7 tetratricopeptide-like repeats (TPRs), an unknown domain (~300 aa) with no clear relationship to any protein families and no confident prediction from FR, and the afore-mentioned Rad18-like CCHC zinc finger. The unknown domain and the zinc finger are missing from the shorter prokaryotic members. Such composition of domains suggests that KIA001018 is a DNA binding protein with endonuclease activity, possibly engaged in DNA repair, with a potential to bind other proteins via TPR domains. The analysis of genomic neighborhood did not reveal any obvious associations with other conserved proteins, which could shed more light on the possible function of KIA001018 and its homologs.

### COG4804/pfam06250 (YhcG family)

COG4804/pfam06250 is represented by the uncharacterized and functionally not annotated protein YhcG from *E. coli*, whose homologs can be found in Prokaryota – mostly in Bacteria, but also in Archaea (a few homologs are found in *Methanosarcinales*) and bacteriophages. The profile HMM analysis confidently identified a relationship (P-value 10^-4^) between the C-terminal domain of YhcG (aa 235–375) and pfam01939 (DUF91)/COG1637, a family of predicted RecB-like nucleases [32]. Both YhcG and DUF91 families exhibit the same orthodox endonuclease motif: E-D-E(I/L/V)K. Nonetheless, our profile HMM analysis indicates that the endonuclease domain of DUF91 is more similar to YraN-like HJRs rather than to the RecB-like nucleases (P-values 10^-6 ^for YraN and 10^-4 ^for DUF91). This is also confirmed by the fold-recognition analysis for YhcG, which indicates the AHJR structures 1hh1 and 1gef/1ipi as possible templates, however with low scores (INBGU: 1gef, score: 7.7; 1hh1, score: 6.08; FUGUE: 1ipi, score: 2.97; 3DPSSM: 1hh1, score: 7.7). COG4804/pfam06250 members possess an additional N-terminal domain (~200 aa), predicted to be mainly α-helical, but lacking any confident matches to known structures, according to fold-recognition servers. The analysis of genomic neighborhood did not reveal any obvious associations of the *yhcG *family with conserved genes. In *Rickettsiae *5 YhcG paralogs could be found in two gene clusters. Only in *Methanosarcina acetivorans *an YhcG homolog was found associated with a type I restriction-modification gene cluster, also with another predicted PD-(D/E)XK superfamily member from COG3586 (a putative endonuclease family, whose members were previously identified [32])

### Pfam06356 (φH_25 family)

Pfam06356 (DUF1064) groups together functionally uncharacterized and not annotated proteins from tailed bacteriophages and bacteria (Proteobacteria and Firmicutes), and is represented by the hypothetical product encoded by ORF25 from *S. aureus *phage φH. The profile HMM analysis identified this family as a relative (P-value from 10^-3 ^to 10^-5^) of YcjD-like proteins – putative members of the PD-(D/E)XK superfamily identified previously [23]. The fold-recognition analysis identified TnsA (1f1z) as one of the possible structural templates (mGenTHREADER: score: 0.302, FUGUE: score: 2.56). The structure of phage T7 Endonuclease I, 1fzr, had slightly better FR scores, but exhibited numerous insertions and deletions in the alignment. Nonetheless, proteins from pfam06356 exhibit a more typical version of the catalytic motif, namely AD-DXK (more similar to T7 Endo I than to TnsA). PSI-BLAST searches using pfam06356 members as queries revealed also a low similarity to the PD-(D/E)XK domain in the Res subunit of Type III restriction-modification systems [41] (alignment only in the endonuclease core region with sequence identity 20% and lower) and to COG3372/pfam5626 (DUF790), an uncharacterized family predicted to belong to the PD-(D/E)XK superfamily in this work, see below. The genomic analysis reveals that pfam06356 members are typically found only in phage genomes or in prophages in bacterial genomes, however, without any strongly conserved neighbors. In only one case, *S. aureus subsp. aureus COL *prophage L54a, the gene coding a putative DNA:m^6 ^A MTase (M.SauCOLORF346P according to the REBASE nomenclature [42]) was found in a putative operon with a pfam06356 member – hypothetical protein SACOL0347, suggesting that together they may form a novel RM system. We speculate that most members of this family may serve a purpose similar to that of phylogenetically unrelated Endonuclease II of phage T4, namely the degradation of the host DNA upon lytic infection as well as production of recombinogenic fragments [43]

### COG5482

COG5482 includes a few functionally uncharacterized and not annotated proteins (~230 aa) mainly from Proteobacteria (only two homologs were found in species from other taxa: *Clostridium acetobutylicum *and *Symbiobacterium thermophilum*). The profile HMM analysis suggested that they are remotely related to AHJRs (P-value ~10^-3^). The fold-recognition analysis has also identified the AHJR structures (1gef/1ipi and 1hh1) as the best templates for modeling of COG5482 sequences, with very good agreement of alignments reported by different servers despite moderate scores (mGenTHREADER: 1hh1, score: 0.543; 1gef, score: 0.413; FUGUE: 1hh1, score: 3.23; 1ipi, score: 3.89; 3DPSSM: 1hh1, score: 3.5; SAM: 1gef/1ipi, scores: 0.17 (0.41). The catalytic domain exhibits the consensus motif: E-CD-ELK. Interestingly, the C-terminal 100 aa was predicted to form a separate domain of the winged helix-turn-helix (wHTH) fold, typically involved in DNA binding [44], which may therefore dictate the target specificity of this putative nuclease. The precise role of these proteins remains to be determined experimentally.

### Pfam06190 (gp51 family)

Pfam06190 (DUF944) groups together very small (~100), functionally uncharacterized and not annotated proteins, exemplified by the gp51 protein from the *Listeria monocytogenes *phage PSA. Homologs of this protein can be found mainly in other phages and in a few bacteria from different taxa, frequently within putative prophages. The profile HMM comparison revealed a remote relationship of Pfam06190 members to AHJRs (P-value ~10^-4^), while sequence analysis revealed an orthodox version of the catalytic motif, (P/S/C)D-EXK. All fold-recognition servers identified PD-(D/E)XK enzymes as the best templates with high scores (e.g. AHJRs (1hh1, 1gef) identified by INBGU, score 25.16, RecU/PrfA recombinase (1y1o, 1rzn) identified by FFAS, score -10.8). Structure prediction suggests that the members of the gp51 family exhibit a minimal form of the central β-sheet, with only four strands, flanked by three α-helices. However, they seem to possess an additional β-hairpin similar to the element, which in RecU forms a dimerization interface (our unpublished analysis of the 1y1o structure). Interestingly, the analysis of genomic neighborhood reveals that gp51 and its homologs are frequently associated with primases or helicases, which suggests they may be involved in DNA replication. We predict that gp51 and its relatives are recombinases involved in resolution of branched intermediates of phage DNA undergoing replication and/or recombination, similarly to T7 endonuclease I.

### COG1395 (PAB2104 family)

COG1395 groups together archaeal sequences of functionally uncharacterized proteins represented by the PAB2104 protein from *Pyrococcus abyssi*, annotated as putative transcription regulators. As might have been expected based on the database annotation, the profile HMM analysis identified with high confidence (P-value ~10^-10^) the helix-turn-helix motif (similar to that one from HTH_3 (Xre) family like proteins: pfam01381, smart00530, cd00093) in the central part of the COG1395 sequences. However, using HHsearch we found that the N – terminal domain is related to the family of AHJRs (pfam01870) (P-value ~10^-6^). The fold-recognition analysis confidently confirmed AHJR (1gef) as the best template for modeling of the N-terminal domain. Preliminary modeling of the N-terminal domain (data not shown) suggests that in PAB2104 and its homologs the catalytic D/E residue from the orthodox (D/E)XK half-motif migrated to the α-helix following the β-strand in which that half-motif was transformed into the "K(I/V)L" form. Interestingly, the profile HMM analysis predicted that the C-terminal domain in COG1395 members may be homologous to the PD-(D/E)XK nucleases as well. This prediction was also supported by the fold recognition analysis, which found AHJR 1hh1 (scores: FFAS: -6.66, INBGU: 13.34, mGenTHREADER: 0.447, SPARKS: -1.74, FUGUE: 4.33, 3DPSSM: 0.061) as the potentially best template structure. However, despite the apparent structural conservation and the presence of hydrophobic residues that confer protein stability, the typical active site is not conserved in this domain (Figure 2). This suggests that the C-terminal domain was generated by intragenic duplication (or fusion of the N-terminal module with a PD-(D/E)XK domain from another source) and then underwent degeneration. Variants of the PD-(D/E)XK domains that lost the active site but presumably retained the ability to bind nucleic acids have been already described (review: [2]).

Thus, COG1395 represents a new family of archaeal putative nucleases with a novel domain architecture: PD-(D/E)XK-HTH-(inactivated)PD-(D/E)XK. It is noteworthy that a related architecture, namely wHTH-PD-(D/E)XK, was observed in the Type IIE REase NaeI, in which the PD-(D/E)XK domain and the wHTH domain, despite the unrelated folds, bind two copies of the same sequence, but only the copy bound to the nuclease domain is cleaved [45]. A PD-(D/E)XK-wHTH fusion (albeit with a reversed order of domains) was also observed in the TnsA transposase [6]. On the other hand, a variant of a tandemly duplicated PD-(D/E)XK domain with a degenerated C-terminal repeat was found in the Sau3AI enzyme [46,47], another Type IIE REase, which also binds as a dimer two copies of the same sequence, but cleaves only the one bound to the pair of "active" N-terminal domains. Tandemly duplicated PD-(D/E)XK domains have been also found in Type IIS restriction enzymes that act as monomers, bind only one asymmetrical DNA target, and use different domains for the cleavage of each strand of the substrate (JMB, JK, and Arvydas Lubys, unpublished data). It will be interesting to study the mode of action of COG1395 members, i.e. whether they act as monomers and dimers, how many copies of the target sequence they bind and whether they act similarly to Type IIE enzymes (like NaeI or Sau3AI) or as nickases, which cleave only one strand of the substrate using the C-terminal active domain.

### COG4127

COG4127 groups together 4 uncharacterized proteins, of which only one, STM4490 from *S. typhimurium*, has been annotated as a predicted restriction endonuclease. PSI-BLAST searches revealed that COG4127 members have numerous homologs in the non-redundant database and are related (E-value ~ 10^-10^) to the Mrr family of REases [18]. The relationship between the "extended" COG4127 family and the Mrr family was confirmed by the profile HMM analysis (P-value ~10^-14^). The fold-recognition analysis identified AHJRs (FFAS: 1hh1, score: -13.5; 1gef/1ipi, score: -7.58; 3DPSSM: 1hh1, score: 3.4; SAM-T02: 1gef/1ipi, score: 0.54) as the best templates for modeling of the C-terminal part of the protein, but failed to suggest a good structural match for the N-terminal domain of a similar size. It is noteworthy that some members of COG4127 contain only the N-terminal domain, but either lack the C-terminal Mrr-like domain or possess another, apparently unrelated domain. Analysis of sequence conservation and predicted secondary structures suggested that the N-terminal domain also resembles the PD-(D/E)XK nucleases, but without the hallmark active site, e.g. like in the afore-mentioned COG1395. However, we were unable to confirm the relationship of the N-terminal domain of COG4127 to known PD-(D/E)XK nucleases either by fold-recognition or by HHsearches. Thus, the structure and function of the N-terminal domain remains to be determined experimentally; the availability of members of COG4127 that already lack the C-terminal domain may be particularly useful.

Interestingly, analyses of genomic context revealed that a few members of COG4127 are associated with putative Type I RM systems (R/S/M.CcrMORF620P in *Caulobacter crescentus*, R/S/M.XorKORF3462P and R/S/M.XorKORF3457P in *Xanthomonas oryzae *and a putative type I RM system in *Methylobacillus flagellatus *not yet included in REBASE) or with a Type III RM system (R/M.DetORF1112P in *Dehalococcoides ethenogenes*). Additionally, another gene cluster from *D. ethenogenes *contains two representatives of COG4127 (one of them contains only the N-terminal domain) and a member of the SfsA family (predicted to be a nuclease in this work). Another COG4127 member from *Azotobacter vinelandii *was found in the neighborhood of the putative Mod subunit and a putative protein annotated as a "virulence protein" (COG3943).

### COG4741

COG4741 consists of functionally uncharacterized proteins mostly from Archaea, of which only one, the hypothetical product of the locus TVN1166 from *T. volcanium *is annotated as "a predicted secreted endonuclease distantly related to AHJRs". The profile HMM analysis confidently (P-value ~10^-5^) identified the relationship of this family to the YraN subfamily of PD-(D/E)XK enzymes [23]. The predicted nuclease domain encompasses 120 C-terminal residues and corresponds to the absolutely minimal core, with only 4 β-strands and 2 α-helices that serve as a scaffold for a well-conserved E-(V/I)D-E(V/I)K motif. The N-terminus reveals a strongly hydrophobic stretch of residues predicted to form a transmembrane helix, which could be either used as a leader peptide to guide the translocation of the nuclease domains through the membrane or it could anchor it to the membrane. Programs for the prediction of transmembrane protein topology HMMTOP [48], TMAP [49], and TMPRED [50] predicted that the nuclease domain has a cytosolic orientation. We speculate that COG4741 members could be released to the environment as toxic agents against other cells, like endonuclease colicins (review: [51]), or be used to guard the cell against the uptake of foreign DNA and/or to cleave the encountered nucleic acids to produce (oligo)nucleotides that can be used by the host.

### COG5321/pfam06319

COG5321/pfam06319 (DUF1052) comprises functionally uncharacterized and not annotated short (~160 aa) proteins found almost exclusively in Proteobacteria. The profile HMM analysis confidently identified their relationship to AHJRs (P-value ~10^-7^). The fold-recognition analysis confirmed the AHJR structures (1gef and 1hh1) as the best templates, however with low scores (data not shown). The reason for this low confidence of fold-recognition could be due to the strong divergence of the C-terminal part of the domain and poor consensus of secondary structure prediction. Nonetheless, COG5321/pfam06319 members exhibit the conserved orthodox AD-E(V/I/C)K motif associated with the characteristic pattern of predicted secondary structures in the N-terminal part of the common fold, which is a strong indication that they are genuine members of the PD-(D/E)XK superfamily, active as nucleases. However, the analysis of genomic neighborhood did not provide any specific clues as to their specific function.

### COG3372/pfam5626

COG3372/pfam5626 (DUF790) is represented by the uncharacterized product of PAB1046 gene from *Pyrococcus abyssi*. Members of this family are found almost exclusively in Archaea and Cyanobacteria. The profile HMM analysis identified their relationship (P-value ~10^-4^) to Pfam06356 (DUF1064) family of putative endonucleases (identified in this work, see above) and a more remote similarity to AHJRs (P-value ~10^-2^). All fold-recognition servers with the exception of FUGUE identified with high scores the structure of bacteriophage T7 Endonuclease I (1fzr, 1m0d) as the best templates for modeling of the C-terminal domain of COG3372 (INBGU: 1fzr, score: 60.96; 1hh1, score: 29.12; 1gef, 19.81; FFAS: 1m0d, score -8.15; mGenTHREADER: 1fzr, score: 0.529; 1hh1, score: 0.459; SAM: 1m0d, score: 1.6*10^-4^; 1fzr, score: 5.4*10^-4^; 1ob8, score: 0.53; SPARKS: 1m0d, score: -3.62; 1ob8, score: -3.28; 1gef, score: -2.8; 1hh1, score: -2.53; FUGUE: 1hh1, score: 4.48; 1fzr, score: 4.26; 1j24, score: 3.01; 3DPSSM: 1fzr, score: 0.53; 1gef, score: 0.6). However, the fold-recognition failed to identify any confident structural matches for the N-terminal domain (size ~250 aa). Analysis of the genomic neighborhood revealed that COG3372 members are usually associated with hypothetical proteins from COG1061 (SSL2: DNA or RNA helicases of superfamily II). This suggests they may be involved in DNA repair or recombination, but does not exclude their potential selfish character.

### gp4 family

The gp4 family is represented by a small (150 aa) protein gp4 from the T4 bacteriophage, annotated as a head completion protein important for the final stages of bacteriophage head assembly process and predicted to be important for the DNA-mediated attachment of independently assembled head and fibers [52]. To our knowledge, only a general functional prediction has been made for this protein and sequences homologous to gp4 have not been grouped into a PFAM or COG family. We identified them by PSI-BLAST, using a Pfam06356 (DUF1064) representative as a query, with a very low E-value of 3.2. The HHsearch analysis carried out for the gp4 family confirmed their relationship to Pfam06356 (DUF1064) (P-value 10^-3^) as well as to COG3372/pfam5626 (P-value 10^-4^) and gp51 families (P-value 10^-3^). Exhaustive PSI-BLAST searches (5 iterations) confidently reported structures of bacteriophage T7 endonuclease I (1fzr, 1m0d) as best templates for the gp4 tertiary structure (E-value = 10^-12^). The consensus of fold-recognition analysis confirmed the prediction pf the PD-(D/E)XK fold, with TnsA structures (1f1z, 1t0f) as well as T7 Endonuclease I (1fzr, 1m0d) reported as the best structural matches. gp4 is positioned in a cluster of genes encoding structural proteins important for the formation of infectious phage particles and to our knowledge has not been reported to interact with the DNA, but to be necessary for the assembly of protein components of the viral particles. The attachment of independently assembled head and fibers is however DNA-dependent (the process does not occur if there is no DNA loaded to the phage head) (review: [52]). Thus, it is possible that gp4 may be involved in determination if the DNA is packed properly or in some yet unknown process involving the DNA cleavage upon the attachment of the tail and fibers to the head, for instance when one end of the packaged DNA descends into the tail.

### PAE1662

PAE1662 from *Pyrobaculum aerophilum *str. IM2 is an uncharacterized, functionally not annotated protein. We found it during exhaustive PSI-BLAST searches using Pfam06356 (DUF1064) representative as a query, with E-value only about 3.4, but also with a hallmark motif E-AD-ELK. We found a characteristic pattern of predicted secondary structure elements associated with the putative nuclease motif, while the fold-recognition servers reported that PAE1662 is similar to several structures of PD-(D/E)XK superfamily members (mGenTHREADER: 1w36C (RecC), score: 0.651; 1hh1, score 0.594; 1ob8, score: 0.538; SAM: 1hh1, score: 3.8; 1t0f, score: 4.2; 1f1z, score 6.3; 3DPSSM: score: 1hh1, score: 1.5). The consensus predictor Pcons selected 1hh1 (AHJR) as a preferred template. Interestingly, PAE1662 is associated with a gene encoding a putative DNA:m^5^C MTase (annotated as M.PaeIMORF1659P in the REBASE database [42]). In this context it is noteworthy that a reciprocal PSI-BLAST search revealed no homologs of PAE1662. Altogether, the association with a DNA MTase, the lack of homologs detectable by database searches and a remote relationship to the PD-(D/E)XK nucleases detected by fold-recognition suggest that PAE1662 is a novel putative type II REase, which to date has not been annotated as such in REBASE.

## Conclusion

We have carried out a sequence/structure profile HMM search using a new method [29] to identify new members of the PD-(D/E)XK superfamily. Our results revealed the presence of this highly diverged nuclease domain in families of proteins implicated in DNA metabolism but with unknown structure (such as a putative recombinase RmuC, DNA competence factor CoiA, a DNA-binding protein SfsA, a large human protein predicted to be a DNA repair enzyme, predicted archaeal transcription regulators, and the head completion protein of phage T4), and in proteins for which no function or structure was assigned to date (such as YhcG, various phage proteins, and novel candidates for restriction enzymes). The initial predictions were validated by protein fold-recognition, leading to preliminary structural models, which were used as platforms for identification of the potential active sites. Thus far, all known members of the PD-(D/E)XK fold were found to be nucleases, mostly acting on the DNA, or at least have been implicated in nucleic acid metabolism. It cannot be excluded that some of the newly reported members may be hydrolases acting on other substrates (e.g. the SfsA protein involved in stimulation of the sugar fermentation), but we speculate that most of them would cleave DNA. The predictions reported in this article will facilitate the search for the possible substrates.

Our predictions contribute to the reduction of "white spaces" on the sequence-structure-function map of the protein universe and will help to jump-start the experimental characterization of the cellular function of these putative nucleases, as well as the molecular mechanisms of their interactions with the DNA. That we identified several members of the PD-(D/E)XK superfamily with very low scores suggests that more strongly diverged members still await discovery. Our analysis has provided a set of new sequence profiles that may be used to search for even more members of this important group of enzymes and will help to select targets for experimental analyses. For instance, it would be interesting to determine high-resolution structures of the presumably "minimal" members of the superfamily (such as the predicted nuclease domain in the C-terminus of the giant protein KIA001018) or proteins with interesting combinations of domains (such as the fusion of predicted active and inactive nuclease domains with the HTH domain in PAB2104). The availability of the complete catalog of nucleases and the knowledge of their mechanisms of action (and interaction) in the cell under different conditions is essential for the complete understanding of mechanisms that govern the evolution and stability of the genome. Our analysis provides a small, but important step towards this aim.

## Methods

### Protein structure prediction

Secondary structure prediction and tertiary fold-recognition was carried out via the GeneSilico meta-server gateway at [53]. Secondary structure was predicted using PSIPRED [54], PROFsec [55], PROF [56], SABLE [57], JNET [58], JUFO [59], and SAM-T02 [60]. Solvent accessibility for the individual residues was predicted with SABLE [57] and JPRED [58]. The fold-recognition analysis (attempt to match the query sequence to known protein structures) was carried out using FFAS03 [61], SAM-T02 [60], 3DPSSM [62], BIOINBGU [63], FUGUE [64], mGenTHREADER [65], and SPARKS [66]. Fold-recognition alignments reported by these methods were compared, evaluated, and ranked by the Pcons server [67]. In the cases, where the active site could not be unambiguously identified from the analysis of alignments, we carried out homology modeling and tried to identify the potential catalytic residues based on structural considerations. Accordingly, fold-recognition alignments to the structures of selected templates were used as a starting point for homology modeling using the "FRankenstein's Monster" approach [68], comprising cycles of model building, evaluation, realignment in poorly scored regions and merging of best scoring fragments. The modeling protocol was essentially identical to that published in [21].

### Construction and comparison of sequence-structure profile HMMs

A set of previously identified members of the PD-(D/E)XK superfamily (Pfu-HJR, GI:5689160; McrC, GI:585466; Mrr, GI:127320; XisH, GI:1613875; YcjD, GI:1175688; A putative nuclease from *Mycobacterium*, GI:15609145; PAB0571, GI:7450881, Yor305wp, GI:6324881; YraN, GI: 1176818; AtF16A14.4, GI:8778385; GP69, GI:465349) were used as seeds in PSI-BLAST [69] searches of the non-redundant (nr) database. For each sequence, the search was carried out in two versions: "conservative", with the expectation (e) value threshold for the retrieval of related sequences set to 10^-6 ^and the maximum number of iterations set to 6, and "aggressive", with the e-value threshold of 10^-2 ^and the maximum number of iterations set to 12. The final blast (blunt-end master-slave) alignments together with the predicted secondary structure were used to generate a set of query profile HMMs using HHmake from the HHsearch package [29]. The profile HMMs corresponding to all COG, KOG [30], PFAM [70], PDB [71], CDD [72] and SMART [73] entries were downloaded from the home site of HHsearch (the Department of Developmental Biology (MPI), ). Comparison of the profile HMMs (sequence+structure) was carried out using HHsearch [29], with default parameters.

## Abbreviations

aa, amino acid(s); bp, base pair(s); nt, nucleotide; e, expectation; REase, restriction endonuclease; ORF, product of an open reading frame,

## Authors' contributions

MF carried out the searches for members of known PD-(D/E)XK families and comparisons of profile HMMs and prepared the initial list of potential new nuclease families. JK validated these predictions using detailed sequence analyses and protein fold-recognition, identified and analyzed "secondary candidates" and participated in writing of the manuscript. JMB provided the set of initial queries for database searches, built the homology models for the validated predictions, interpreted all data and drafted the manuscript. All authors have read and accepted the final version of the manuscript.

## Supplementary Material

Additional File 1 **An archive file in the zip format, which includes Hidden Markov Models of sequence & secondary structure profiles of PD-(D/E)XK nuclease families analyzed in this article (those known used as queries and newly identified ones). **HMMs are in the format of the HHsearch program. The same data are available for download from the authors' FTP server: Click here for file
